# Selective enrichment of high-affinity clade II N_2_O-reducers in a mixed culture

**DOI:** 10.1093/ismeco/ycaf022

**Published:** 2025-02-05

**Authors:** Michele Laureni, Francesc Corbera-Rubio, DaeHyun Daniel Kim, Savanna Browne, Nina Roothans, David G Weissbrodt, Karel Olavaria, Nadieh de Jonge, Sukhwan Yoon, Martin Pabst, Mark C M van Loosdrecht

**Affiliations:** Department of Biotechnology, Delft University of Technology, Van der Maasweg 9, Delft, HZ NL- 2629, The Netherlands; Department of Biotechnology, Delft University of Technology, Van der Maasweg 9, Delft, HZ NL- 2629, The Netherlands; Department of Civil and Environmental Engineering, Korea Advanced Institute of Science and Technology, Daehakro 291, KAIST, Daejeon 34141, South Korea; Department of Civil and Environmental Engineering, University of California, Berkeley, CA, USA; Department of Biotechnology, Delft University of Technology, Van der Maasweg 9, Delft, HZ NL- 2629, The Netherlands; Department of Biotechnology, Delft University of Technology, Van der Maasweg 9, Delft, HZ NL- 2629, The Netherlands; Department of Biotechnology and Food Science, Norwegian University of Science and Technology, Sem Sælands vei 8, Trondheim 7034, Norway; Department of Biotechnology, Delft University of Technology, Van der Maasweg 9, Delft, HZ NL- 2629, The Netherlands; Department of Chemistry and Bioscience, Aalborg University, Fredrik Bajers Vej 7H, Aalborg DK-9220, Denmark; Department of Civil and Environmental Engineering, Korea Advanced Institute of Science and Technology, Daehakro 291, KAIST, Daejeon 34141, South Korea; Department of Biotechnology, Delft University of Technology, Van der Maasweg 9, Delft, HZ NL- 2629, The Netherlands; Department of Biotechnology, Delft University of Technology, Van der Maasweg 9, Delft, HZ NL- 2629, The Netherlands

**Keywords:** nitrous oxide, N_2_O respiration, clade II NosZ, cobalamin, vitamin b12, N_2_O cytotoxicity, mixed culture, non-axenic enrichment

## Abstract

Microorganisms encoding for the N_2_O reductase (NosZ) are the only known biological sink of the potent greenhouse gas N_2_O and are central to global N_2_O mitigation efforts. Clade II NosZ populations are of particular biotechnological interest as they usually feature high N_2_O affinities and often lack other denitrification genes. We focus on the yet-unresolved ecological constraints selecting for different N_2_O-reducers strains and controlling the assembly of N_2_O-respiring communities. Two planktonic N_2_O-respiring mixed cultures were enriched at low dilution rates under limiting and excess dissolved N_2_O availability to assess the impact of substrate affinity and N_2_O cytotoxicity, respectively. Genome-resolved metaproteomics was used to infer the metabolism of the enriched populations. Under N_2_O limitation, clade II N_2_O-reducers fully outcompeted clade I affiliates, a scenario previously only theorized based on pure-cultures. All enriched N_2_O-reducers encoded and expressed the sole clade II NosZ, while also possessing other denitrification genes. Two *Azonexus* and *Thauera* genera affiliates dominated the culture, and we hypothesize their coexistence to be explained by the genome-inferred metabolic exchange of cobalamin intermediates. Under excess N_2_O, clade I and II populations coexisted; yet, proteomic evidence suggests that clade II affiliates respired most of the N_2_O, *de facto* outcompeting clade I affiliates. The single dominant N_2_O-reducer (genus *Azonexus*) notably expressed most cobalamin biosynthesis marker genes, likely to contrast the continuous cobalamin inactivation by dissolved cytotoxic N_2_O concentrations (400 μM). Ultimately, our results strongly suggest the solids dilution rate to play a pivotal role in controlling the selection among NosZ clades, albeit the conditions selecting for genomes possessing the sole *nosZ* remain elusive. We furthermore highlight the potential significance of N_2_O-cobalamin interactions in shaping the composition of N_2_O-respiring microbiomes.

## Introduction

Nitrous oxide (N_2_O) is a potent greenhouse gas, with a global warming potential almost 300 times higher than CO_2_, and it is the predominant ozone-depleting substance in the atmosphere [1]. N_2_O emissions have been increasing globally at a rate of 17 Tg N y^−1^ over the last decade [2], and this rise is expected to continue if no mitigation efforts are put in place [1]. Most of the produced N_2_O results from microbially mediated reactions in managed and engineered ecosystems characterized by high nitrogen loads, such as agricultural soils and wastewater treatment plants (WWTPs) [3, 4]. In-depth understanding of the microbiology underlying N_2_O production and consumption is paramount in emission mitigation endeavors.

N_2_O is produced primarily as a by-product of the first nitrification step, namely the biological oxidation of ammonia (NH_3_) to nitrite (NO_2_^−^), or during incomplete denitrification [5, 6]. Denitrification is the sequential reduction of dissolved nitrate (NO_3_^−^) and NO_2_^−^ to dinitrogen gas (N_2_), with nitric oxide (NO) and N_2_O as obligate free intermediates [7]. Each reduction step is catalyzed by distinct enzymes and can function as an independent energy-conserving reaction [4, 7, 8]. Denitrification can be performed by a single microorganism (termed “generalist”) or a consortium of cooperating “specialists”, each of them performing one or few nitrogen oxides reductions [9]. As such, denitrification can act as a source or a sink of N_2_O depending on the genetic and metabolic repertoire of the microbial community members, and the environmental conditions [10, 11]. Importantly, the reduction of N_2_O to N_2_ catalyzed by the N_2_O-reductase (NosZ), the last step of the denitrification pathway, is the only known biological N_2_O sink [4].

The ability to reduce N_2_O is taxonomically widely distributed among diverse microbial groups [12], and the *nosZ* gene evolved in two phylogenetically distinct lineages, namely clade I and II [4]. Interestingly, clade II NosZ appears to be more often encoded in genomes of non-denitrifying (specialist) N_2_O-reducers, namely microorganisms lacking other denitrifying enzymes that represent possible net N_2_O sinks [4, 13, 14]. Limited pure culture-based observations suggests that clade II organisms feature higher affinities for N_2_O than clade I populations, albeit at usually lower maximum specific growth rates [15, 16]. On these grounds, clade II N_2_O-reducers hold the potential to further curb emissions by reducing residual dissolved N_2_O concentrations, irrespective of their denitrifying capacity.

Denitrifying communities dominated by N_2_O-reducing specialists [17] and rich in clade II affiliates are ubiquitous [9, 12, 18–23]. The abundance of clade II populations has been reported to inversely correlate with N_2_O emissions from soils [10, 24, 25]. High relative abundances of clade II N_2_O-reducers have been obtained in lab-grown biofilms [15, 26, 27]. In their seminal chemostat work, Conthe and co-workers [28, 29] reported a marked increase in clade II *nosZ* gene abundance when the dilution rate was decreased from 0.086 to 0.027 h^−1^. Yet, to date, no enrichment has resulted in the selection of sole clade II organisms. Different traits such as the preferred electron donor [30], the sensitivity to other electron acceptors like NO_3_^−^ [31] and O_2_ [30], or the required presence of other nitrogen oxides [32] may also contribute to clades selection. N_2_O itself may result in direct cytotoxicity, hindering microbial growth by selectively inactivating vitamin B_12_ [33], with implications on community assembly that remain largely unknown. Resolving the ecological constraints controlling the selection of different N_2_O-reducers would greatly benefit both biotechnological designs and our understanding of the biosphere response to a rapidly changing climate.

We postulate that the high solids dilution rates used in previous studies prevented the selection of clade II N_2_O-reducers [28, 29]. To verify our hypothesis, two N_2_O-respiring mixed cultures were enriched under limitation of either electron donor (acetate) and electron acceptor (N_2_O), and an ultrafiltration membrane was used to impose a solids dilution rate five times lower than ever tested. We employed continuous chemostat reactors to select, by design, for organisms with the highest affinity for the growth-limiting substrate [34], enabling direct comparisons without requiring the kinetic characterization of individual strains. The taxonomy and metabolic potential of the dominant community members at steady-state were identified via genome-resolved metagenomics. Shotgun metaproteomics was used to estimate the relative biomass contribution and infer the actual metabolism of each organism. Under N_2_O-limiting conditions we successfully cultivated a mixed culture in which all N_2_O-reducers encoded and expressed the sole clade II NosZ, while still the conditions were not selective enough for specialist N_2_O-reducers. Additionally, metagenomic and metaproteomic data suggest a potential role of vitamin B_12_ cross-feeding in the assembly of N_2_O converting communities, which has not been previously reported.

## Materials and methods

### Continuous enrichments

**Figure 1:**
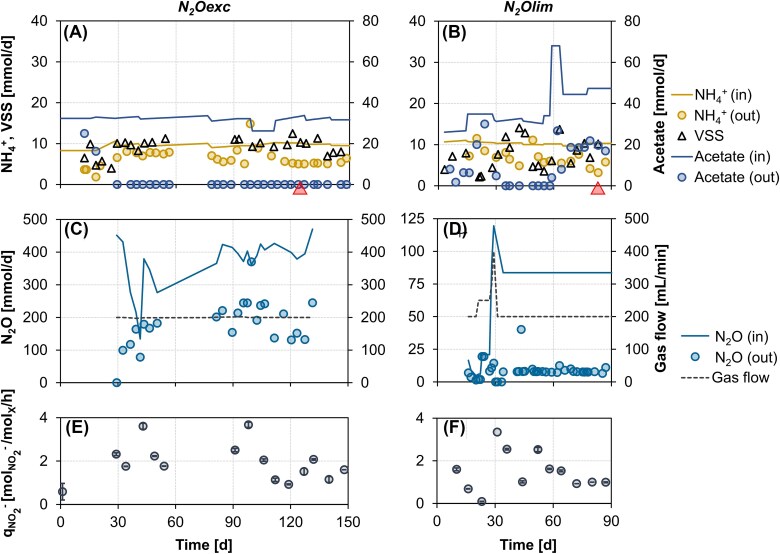
Overall and biomass specific performance of the two cultures enriched under excess (*N_2_Oexc*) and limiting (*N_2_Olim*) availability of nitrous oxide as sole electron donor. (A and B) Influent and effluent acetate and ammonium rates, and biomass production rate. *Full triangle*: day of biomass sampling for metagenomic and metaproteomic analysis. (C and D) influent and effluent N_2_O rates, and imposed gas flow. (E and F) *Ex-situ* maximum biomass specific NO_2_^−^ consumption rate.

Two identical glass, continuous-flow membrane bioreactors (MBR) with a working volume of 2 L (Applikon, Delft, the Netherlands) were operated with acetate and N_2_O as sole electron donor and acceptor, respectively. To allow for direct comparisons, the set-up was identical to the one used by Conthe and co-workers [29]. The sole difference was the use in our set-up of a custom-made, submerged ultrafiltration membrane module [35] to uncouple the solids retention time (SRT) and the hydraulic retention time (HRT). In both MBRs, the HRT was maintained at 2.8 ± 0.3 d. The SRT was set at 6.9 ± 1.0 d, equivalent to a biomass dilution rate (D) of 0.006 ± 0.001 h^−1^ (Table 1). The imposed D was lower than the growth rate reported for clade II *nosZ*-possessing *Anaeromyxobacter dehalogenans* 2CP-C (0.0076 h^−1^), the slowest-growing N_2_O-reducing bacterium examined by [16], and the minimum dilution rate (0.027 h^−1^) that has been applied in previous non-axenic N_2_O-reducing chemostat works [29]. The temperature was controlled at 20 ± 1°C, and the pH was maintained at 7.0 ± 0.1 with 1 M HCl and 1 M NaOH. Stirring at 750 rpm was ensured by two six-bladed flat turbines. Two independent 5850S mass-flow controllers (Brooks, Philadelphia, PA) were used in each MBR to mix pure N_2_O and N_2_, and achieve the target overall gas flow and N_2_O concentration (Fig. 1). The carbon and nitrogen sources were provided separately. The two mineral media contained per liter: 90.5 mmol CH_3_COONa·3H_2_O or 26.4 mmol NH_4_Cl (anabolic nitrogen source), respectively, additionally to 7.4 mmol KH_2_PO_4_, 2.1 mmol MgSO_4_·7H_2_O, 0.5 mmol NaOH, 2 mg yeast extract and 2.5 ml of trace element solution [36]. The first MBR operated under acetate limitation and N_2_O excess (hereafter referred to as *N_2_Oexc*) was inoculated directly with activated sludge from the Harnaschpolder WWTP (Delft, the Netherlands). Excess N_2_O conditions, defined as no detectable acetate in the effluent, were achieved with influent acetate and N_2_O loads of 31.6 ± 2.0 and 382 ± 25 mmol/d, respectively, after a 30-days start-up phase. The second MBR operated under N_2_O limitation (*N_2_Olim*) was inoculated with biomass collected from *N_2_Oexc* after 98 days of operation. N_2_O limiting conditions in *N_2_Olim* were reached on day 60, and maintained until day 95 at influent acetate and N_2_O loads of 46.1 ± 1.5 and 84 ± 2.1 mmol/d, respectively (Fig. 1). The residual acetate concentration in *N_2_Olim* was 23.7 ± 5.5 mmol/L. Occasional growth on the MBR walls and membrane was cleaned on a weekly basis.

**Table 1:** Biomass, ammonium, and N_2_O stoichiometric yields of the *N_2_Oexc* and *N_2_Olim* enrichments. Values were calculated based on the measured consumption and production rates after data reconciliation (days 100–150 for *N_2_Oexc* and 60–100 for *N_2_Olim*; Table S1), and standard deviations were estimated by propagating the relative error of each measured rate. The yields obtained by [29] at higher dilutions with an identical set-up, used for the estimation of the biomass-specific substrate consumption rate for maintenance, are also presented. Stoichiometrically, 4 moles of N_2_O are required to respire 1 mole of acetate.

**D**	**N**_**2**_ **O/Ac (inf.)**	**Y**_**X/Ac**_	**Y**_**X/N2O**_	**Y**_**N2O/Ac**_	**Y**_**NH4/X**_	
**h** ^**−1**^	**mol/mol**	**mol/mol**_**C**_	**mol/mol**	**mol/mol**	**mol/mol**	
0.006 ± 0.001	12.8 ± 1.5	0.15 ± 0.03	0.09 ± 0.02	3.42 ± 0.35	0.41 ± 0.15	**N**_**2**_ **Oexc**
0.028 ± 0.001	15.9	0.25 ± 0.02	0.16 ± 0.01	3.06 ± 0.2	0.28 ± 0.02	Conthe et al. 2018c
0.089 ± 0.003	26.1	0.27 ± 0.01	0.17 ± 0.01	3.1 ± 0.17	0.28 ± 0.01	Conthe et al. 2018c
0.006 ± 0.001	1.8 ± 0.1	0.24 ± 0.04	0.15 ± 0.03	3.1 ± 0.15	0.44 ± 0.13	**N**_**2**_ **Olim**
0.027 ± 0.001	2.3	0.32 ± 0.04	0.22 ± 0.04	2.86 ± 0.51	0.26 ± 0.01	Conthe et al. 2018c
0.086 ± 0.003	2.7	0.33 ± 0.03	0.26 ± 0.02	2.49 ± 0.29	0.25 ± 0.02	Conthe et al. 2018c

### Nitrite reduction batch tests

We aimed to quantify the long-term dynamics of the denitrification potential of the enrichments respiring exclusively N_2_O. To this end, the maximum biomass-specific nitrite reduction rate (q_NO2-_; mmol_NO2-_·mmol_X_^−1^·h^−1^) was used as denitrification proxy and quantified in batch. One liter of fresh reactor effluent was collected under anoxic conditions, via continuous N_2_-sparging of the effluent collection vessel with N_2_ gas, and centrifuged at 4200 rpm for 20 min at room temperature. The recovered pellet was re-suspended in N_2_-sparged base mineral medium without ammonium or acetate, with the same composition as detailed above. Based on the biomass concentration measured in the chemostat, the cells were re-suspended to a concentration of 0.9 ± 0.2 g_VSS_·L^−1^. Subsequently, 50 ml of cell suspension were aliquoted in 112 ml serum bottles. The bottles were sealed with rubber stoppers, and anoxic conditions were achieved by flushing with N_2_ for 20 min. After overnight incubation, appropriate volumes of anoxic stock-solutions of NH_4_Cl, CH_3_COONa·3H_2_O, and NaNO_2_ were added to reach the target initial concentrations of 2.1 mmol NH_4_^+^·L^−1^, 1.2 mmol CH_3_COO^−^·L^−1^, and 1.1 mmol NO_2_^−^·L^−1^. The frequency of bulk liquid sampling (4 mL) depended on biomass activity. Samples were immediately centrifuged for 5 min (4200 rpm, 4°C), and the supernatant was kept for further analysis. The volumetric NO_2_^−^ consumption rate was determined by calculating the slope of the linear regression of at least four nitrate concentration points measured during each batch. Average and standard deviations were obtained from replicate bottles. Subsequently, the biomass-specific reduction rates were calculated by dividing the volumetric rates by the corresponding experimentally determined biomass concentration. The resulting standard deviations were calculated based on the average and standard deviations of the volumetric rates and biomass concentrations using linear error propagation. Initial and final acetate concentrations were measured to confirm non-limiting conditions. All tests were performed in an Incubator Hood TH30 (Edmund Bühler GmbH, Bodelshausen, Germany) at 20°C and 150 rpm. Negative controls with autoclaved biomass were also included. The pH remained in the range 7–8 without external control.

### Analytical procedures and calculations

Samples for influent and effluent supernatant analysis were centrifuged at 4°C and 13 000 rpm for 5 min, stored at −20°C, and analysed within less than 6 days. Bulk NH_4_^+^, NO_2_^−^ and NO_3_^−^ concentrations were measured spectrophotometrically with the Gallery™ Discrete Analyzer (Thermo Fisher Scientific). Acetate concentration was measured by high-performance liquid chromatography (Vanquish Core HPLC, Thermo Fisher Scientific) using an Aminex HPX-87H column (300 × 7.8 mm; Bio-Rad, Waters Chromatography B.V.), calibrated with standard solutions ranging from 0 to 250 mM. In- and off-gas N_2_O concentrations were continuously monitored online by a Rosemount NGA 2000 off-gas analyser (Emerson). Before reaching the analyser, the off-gas was dried in a condenser operated with water at 4°C using a cryostat bath (Lauda). N_2_O concentrations in the in- and off-gas of *N_2_Oexc* exceeded the off-gas analyser range, thus weekly grab-samples were measured with a CP-3800 gas chromatograph (Varian). The concentrations of total and volatile suspended solids (TSS, VSS) in the mixed liquors were determined according to standard methods [37], and an average biomass composition of CH_1.8_O_0.5_N_0.2_ [38] was assumed for downstream molar calculations. All stoichiometric yields (Table 1) were calculated as the ratio of the corresponding steady-state volumetric conversion rates (Table S1) after data reconciliation using the software Macrobal [39]. The resulting standard deviations were calculated based on the average and standard deviations of the individual volumetric rates using linear error propagation. The biomass-specific acetate consumption rate for maintenance was estimated by linear regression of the growth yields on acetate over the D range covered by this study and [29] (Fig. S1). Dissolved N_2_O concentrations were estimated from the average off-gas N_2_O concentrations with a Henry’s constant of 2.4·10^−4^ mmol/(L·Pa) [40].

### Metagenomics

*Metagenome sequencing.* Samples for metagenomic analysis were taken on days 123 (*N_2_Oexc*) and 83 (*N_2_Olim*). DNA was extracted using the DNeasy UltraClean Microbial Kit (Qiagen, The Netherlands). Library preparation and metagenomic sequencing were performed by Novogene Ltd. (Hongkong, China) on HiSeq platform (Illumina Inc., CA). Libraries were generated from 1 μg DNA per sample using the NEBNext Ultra DNA Library Prep Kit (NEB #E7645, USA) following the manufacturer’s instructions. DNA was fragmented by sonication to a size of 350 bp, and DNA fragments were end-polished, A-tailed, and ligated with a full-length adapter for further PCR amplification. PCR products were purified (AMPure XP system), and libraries were quantified using real-time PCR after size distribution analysis using an Agilent 2100 Bioanalyzer. Index-coded samples were clustered with a cBot Cluster Generation System according to the manufacturer’s instructions. Ultimately, libraries were sequenced to generate 150 bp paired-end reads (HiSeq sequencing platform, Illumina Inc., US). *Metagenome assembly and binning.* The raw shotgun metagenome reads were screened using Trimmomatic v0.36 with the parameters set as follows: *LEADING:3 TRAILING:3 SLIDINGWINDOW:4:15 MINLEN:70* [41]. The trimmed reads were assembled de novo into contigs using metaSPAdes v3.14.0 with default parameters [42]. The trimmed reads were mapped back onto the assembled contigs using the *mem* algorithm implemented in BWA v0.7.17, and the mapping data were sorted using SAMtools v1.10 [43, 44]. The coverage of each contig was calculated using *jgi_summarize_bam_contig_depths* command implemented in MetaBat2 with the parameters *minContigLength* and *minContigDepth* set to 2000 and 2, respectively [45]. These coverage data were used as the input to MetaBat2 for binning the contigs into metagenome assembled genomes (MAG) [45]. CheckM v1.1.2 software was implemented to assess the quality of the bins by computing their completeness and contamination (*lineage_wf* command) [46]. MAGs potentially belonging to a single organism were identified and subsequently merged (*merge* command) after manually inspecting their marker gene sets and coverage information [46]. The bins were subsequently refined using refineM, removing the contigs violating consistency in terms of contig statistics (computed with *scaffold_stats* command) and/or taxonomic affiliations of the protein-coding sequences within [47]. Protein-coding sequences were also predicted with refine using the *call_genes* command and their taxonomic affiliation was inferred using the *taxon_profile* command with the GTDB database release 80 as reference. Subsequently, inconsistent contigs were identified with the *taxon_filter* and removed. After filtering out all potential outlier contigs, the bins were further filtered with the completeness and contamination thresholds set to 75% and 5%, respectively. *Metagenome annotation.* The taxonomy affiliations of the refined MAGs were inferred using GTDB-tk v2.3.2 with GTDB database release 214 [48]. To estimate the relative abundances of the organisms represented by the MAGs, the quality-trimmed raw reads were mapped onto the contigs in the finalized MAGs using BWA MEM v0.7.17 with default parameters [43]. The alignment files were sorted with SAMtools v1.10, and the coverages of the contigs in the MAGs were calculated using *coverage* command of checkM v1.1.2 [46]. The relative abundance of each MAG was calculated using *profile* command and represented as the percentage of total reads mapped to the contigs in the MAG, normalized by the total size of the contigs in the MAG. The potential coding sequences within each MAG were annotated using MetaErg v1.2.0 with Pfam, TIGRFAMS, Metabolic-hmms, SwissProt, and RefSeq databases as reference [49]. Annotation was supplemented with Ghostkoala [50]. The functions, domain structures, KEGG Orthology (KO) numbers, Gene Orthology numbers (GO), and Enzyme Commission numbers (EC) were included in the annotation. The annotation of the two *nosZ* clades was manually refined on Interpro [51] based on the twin-arginine translocation (clade I, IPR006311) or the general secretory (clade II, IPR026468) pathway-specific signal peptides [52]. The cobalamin-dependent epoxyqueuosine reductase *queG* (EC 1.17.99.6), and its cobalamin-independent functional homolog *queH* [53], were differentiated phylogenetically using annotated *queG* and *queH* sequences from UniProtKB [54]. The same approach was used to differentiate the two nucleotide loop assembly marker genes for cobalamin production, *cobP*, *cobU*, and *cobY*. The software Anvi’o v.8 was used to align concatenated ribosomal genes identified across MAGs, and the phylogenetic tree was built from the alignment using FastTree (Fig. S2).

### Mass spectrometry based proteomics [55, 56]

*Protein extraction and proteolytic digestion. Protein extraction.* Approximately 25 mg of biomass (wet weight) was collected in an Eppendorf tube and solubilized in a suspension buffer consisting of 175 μL B-PER reagent (Thermo Scientific) and 175 μL TEAB buffer (50 mM TEAB, 1% (w/w) NaDOC, adjusted to pH 8.0). Next, 0.1 g of glass beads (acid-washed, approximately 100 μm in diameter) were added and the cells were disrupted using 5 cycles of bead beating on a vortex for 1 min, followed by cooling on ice for 30 s. Then, a freeze/thaw step was performed by freezing the suspension at −80°C for 20 min, and thawing it while shaking in an incubator. The cell debris was pelleted at 14000 × g for 10 min while being cooled at 4°C. The supernatant was transferred to a new Eppendorf tube. *Protein precipitation.* The protein was precipitated by adding 1 volume of TCA (trichloroacetic acid) to 4 volumes of the supernatant. The solution was then incubated at 4°C for 10 min and pelleted again at 14000 × g for 10 min. The resulting protein pellet was washed twice with 200 μL of ice-cold acetone. *Trypsin digestion.* The protein pellet was dissolved in 200 mM ammonium bicarbonate containing 6 M urea to achieve a final concentration of approximately 100 μg/μL. To 100 μL of the protein solution, 30 μL of a 10 mM DTT solution was added and incubated at 37°C for 1 h. Next, 30 μL of a freshly prepared 20 mM IAA solution was added and incubated in the dark for 30 min. The solution was then diluted to a concentration of <1 M Urea using a 200 mM ammonium bicarbonate buffer, and an aliquot of approximately 25 μg of protein was digested with Trypsin (Promega, Trypsin to protein ratio of 1:50) at 37°C overnight. *Solid phase extraction.* The peptides were desalted using an Oasis HLB 96-well plate (Waters) as per the manufacturer's instructions. The purified peptide eluate was then dried using a speed vac concentrator. *Large-scale shotgun proteomics and label-free quantification.* An aliquot containing approximately 250 ng protein digest was analysed in duplicates using an one-dimensional shotgun proteomics approach. Each duplicate was injected twice. Briefly, the samples were analysed using a nano-liquid-chromatography system consisting of an EASY nano-LC 1200, equipped with an Acclaim PepMap RSLC RP C18 separation column (50 μm × 150 mm, 2 μm), and an QE plus Orbitrap mass spectrometer (Thermo Scientific, Germany). The flow rate was maintained at 350 nL/min. Following sample loading and a wash step at 5% B, a linear gradient was performed, first to 25% B over 88 min, and further to 50% B over additional 25 min. Finally, the separation column was back equilibration to starting conditions. Solvent A was H_2_O containing 0.1% formic acid, and solvent B consisted of 80% acetonitrile in H_2_O and 0.1% formic acid. The spectrometer was operated in the data dependent acquisition mode, thereby measuring peptide signals from the 385–1250 m/z range at 70 K resolution with the AGC target of 3e6 and max IT of 100 ms. The top 10 signals were isolated at a window of 2.0 m/z and fragmented using a NCE of 28. Fragments were acquired at 17 K resolution, at an AGC target of 5e4 and 100 ms max IT. *Database searching, comparison of conditions, and data visualization.* Data were analysed using a database of *in silico* translated gene-coding sequences obtained from shotgun metagenome experiments of the analysed enrichment cultures (as described above) with PEAKS Studio 10 (Bioinformatics Solutions Inc, Canada). Database searching was performed by allowing for 20 ppm parent ion mass error tolerance and 0.02 m/z fragment ion mass error tolerance and allowing of up to 2 missed cleavages. Carbamidomethylation was set as fixed modification and methionine oxidation and N/Q deamidation were considered as variable modifications. Peptide spectrum matches were filtered against 1% false discovery rate (FDR) and protein identifications with ≥2 unique peptides were considered as significant identifications. The relative abundance of each protein is presented in terms of normalized spectral counts, defined as spectral counts per protein divided by its molecular weight, and divided by the sum of spectral counts of the specific injection. Only proteins identified in at least two out of four injections were included in the analysis, and the average of their normalized spectral counts in the different injections is taken as proxy for their relative abundance. RStudio v2023.03.1 [57] with R v4.3.0 [58] was used for data analysis and visualization.

### Amplicon sequencing

Biomass samples were collected on a weekly basis for 16S rRNA gene amplicon sequencing. The protocols for DNA extraction, sequencing, and data processing and analysis, are detailed in Fig. S3 in the Supporting Information. The resulting microbial composition profiles at Family level are also presented therein.

## Results and discussion

### Reduced biomass growth under continuous N_2_O excess

Two N_2_O-respiring mixed cultures were successfully enriched in a chemostat over multiple generations with acetate and N_2_O as sole electron donor and acceptor, respectively (Fig. 1; Table S1). Acetate (*N_2_Oexc*) and N_2_O (*N_2_Olim*) limiting conditions were imposed to assess the impact of substrate affinity on community assembly at low dilution rates. Specifically, the imposed dilution rate was almost five times lower than previously applied in non-axenic N_2_O-reducing chemostat works [29]. Both enrichments featured lower biomass growth yields on N_2_O (Y_X/N2O_) and acetate (Y_X/Ac_) compared to those obtained by [29] with an identical set-up and comparable operating conditions with the exception of the ultrafiltration membrane used in this study (Table 1). The observed lower yields are consistent with greater relative allocation of resources to maintenance at lower growth rates. The stoichiometric consumption of 3.1–3.4 moles N_2_O per mole acetate consumed was slightly higher than the values obtained by [29] (Table 1). The yields of ammonium consumed per biomass produced (Y_NH4/X_) were higher than expected for the commonly assumed empirical biomass composition (CH_1.8_O_0.5_N_0.2_ [38]), yet comparable to the ones of the *Pseudomonas stutzeri* JM300 strain grown on N_2_O and acetate [29].

Importantly, the growth yields on both N_2_O and acetate were substantially lower in *N_2_Oexc* than in *N_2_Olim* (Table 1). This is in agreement with [29], and likely relates to the potential cytotoxic effects of N_2_O [33, 59, 60]. Based on average off-gas N_2_O concentrations, the estimated dissolved N_2_O concentration in *N_2_Oexc* was 382 ± 25 μM, exceeding by far the concentration at which cytotoxic effects have been previously reported [60]. The estimated biomass-specific acetate consumption rate for maintenance was 2.4-fold higher under N_2_O excess (0.019 ± 0.001 C-mol_Ac_·mol_X_^−1^·h^−1^) than when N_2_O was limiting (0.008 ± 0.001 C-mol_Ac_·mol_X_^−1^·h^−1^). This further supports the negative impact of N_2_O on growth.

### *Rhodocyclaceae*-dominated N_2_O-respiring enrichments

To resolve the identity and metabolic potential of the enriched organisms we sequenced the metagenomes of the two steady-state cultures. A total of 75.2·10^6^ and 64.5·10^6^ reads (*N_2_Oexc* and *N_2_Olim*, respectively) were obtained after quality filtering, and their assembly yielded 6550 and 7001 contigs (> 2000 bp) with N50 values of 18.8·10^3^ and 12.6·10^3^ bp. Contigs were binned, resulting in 10 and 17 metagenome-assembled genomes (MAGs), with completeness >75% and contamination <5% (Table 2). The recovered MAGs accounted for 94.5 and 74.1% of the quality-filtered reads in *N_2_Oexc* and *N_2_Olim*, respectively. The obtained binning coverage indicates that the recovered MAGs represent the majority of the two microbiomes. In terms of relative reads abundance, AZO_exc (*N_2_Oexc*) and AZO_lim (*N_2_Olim*) were the dominant MAGs, and accounted for about 83.2 and 67.1% of the reads in the respective reactors. Low quality MAGs were grouped with the unbinned reads (Table 2).

**Table 2:** Genomic and proteomic characteristics of the recovered MAGs. MAGs marked with (*) affiliate with the *Rhodocyclaceae* family. MAGs marked with (^**‖**^) or (^**‡**^) share a genome-aggregate average nucleotide identity (ANI) > 99%.

		**Metagenome**								**Metaproteome**		
	**Bin ID**	**Complet.**	**Contam.**	**Bin size** **[Mbp]**	**N50** **[bp]**	**GC** **[%]**	**Mapped reads**	**Mapped** **reads [%]**	**Predicted** **proteins**	**Detected** **proteins**	**Rel. abund.** **spectra [%]**	**Taxonomy**
***N***_***2***_ ***Oexc***	AZO_exc*	97.6	0.0	2.7	171 993	59.8	62 591 220	83.2	2614	1077	75.8	Genus *Azonexus*
	**Bin.2_exc**	94.5	0.4	3.2	49 531	60.5	1 470 411	2.0	3009	143	5.4	Genus *Phaeovulum*
	**Bin.24_exc**	97.5	0.5	3.3	33 922	59.3	1 160 565	1.5	3023	117	5.1	Genus *Pseudorhodobacter*
	**Bin.15_exc**	98.2	0.0	3.5	133 854	46.6	4 181 484	5.6	3299	98	3.7	Genus *Bdellovibrio*
	**Bin.3_exc**	77.8	1.8	3.1	20 050	65.3	328 268	0.4	3014	20	0.8	Genus *Aquamicrobium*
	**Bin.12_exc**	98.9	0.1	3.1	74 431	33.7	245 691	0.3	2787	6	0.3	Species *Flavobacterium filum*
	**Bin.18_exc** ^**‖**^	99.5	0.0	2.7	81 605	40.4	547 907	0.7	2424	8	0.3	Genus *Moheibacter*
	**Bin.16_exc**	99.0	0.0	2.5	57 459	37.3	213 672	0.3	2262	8	0.2	Genus *Kaistella*
	**Bin.17_exc**	99.0	0.0	2.9	65 957	39.2	241 612	0.3	2480	3	0.1	Genus *Ferruginibacter*
	**Bin.9_exc** ^**‡**^	95.8	0.5	2.5	13 365	35.0	131 267	0.2	2281	*-*	*0.0*	Genus *Ferruginibacter*
	***Unbinned***	*-*	*-*	26.4	5341	*-*	4 099 244	5.5	27 394	133	8.4	-
												
***N_2_Olim***	AZO_lim*	87.3	0.0	2.7	32 281	63.5	43 300 280	67.1	2492	648	50.8	Genus *Azonexus*
	THA_lim*	100.0	0.7	4.2	38 798	69.5	1 247 092	1.9	3747	181	13.5	Species *Thauera phenylacetica*
	**Bin.14_lim** ^**‖**^	77.2	0.1	2.0	4921	40.5	78 531	0.1	2090	43	2.1	Family *Weeksellaceae*
	**Bin.21_lim** ^**‡**^	99.5	0.3	2.6	104 725	35.2	520 671	0.8	2170	60	1.8	Genus *Ferruginibacter*
	**Bin.6_lim**	75.4	1.8	2.5	24 195	48.9	243 575	0.4	2366	17	0.7	Species *Seleniivibrio woodruffii*
	**Bin.24_lim**	78.0	1.1	1.3	57 096	24.0	486 035	0.8	1137	12	0.5	Phylum *Patescibacteria*
	Bin.20._lim*	76.9	0.9	3.1	6309	66.0	141 837	0.2	3184	7	0.4	Species *Azovibrio restrictus*
	**Bin.26_lim**	89.8	3.9	2.4	13 434	61.7	134 723	0.2	2332	1	0.2	Genus *Pseudoflavonifractor*
	**Bin.11_lim**	93.0	1.6	3.9	17 980	43.9	214 463	0.3	3203	6	0.1	Order *Bacteroidales*
	**Bin.2_lim**	93.5	0.1	1.7	11 915	50.4	233 247	0.4	1697	4	0.1	Family *Anaerovoracaceae*
	**Bin.8_lim**	78.5	2.8	2.6	4471	38.0	101 235	0.2	2495	3	0.1	Genus *Niabella*
	**Bin.10_lim**	93.7	1.1	2.9	42 970	33.1	262 567	0.4	2266	3	0.1	Order *Bacteroidales*
	**Bin.1_lim**	76.2	0.1	1.1	4590	37.2	43 318	0.1	1208	1	0.1	Genus *Paracholeplasma*
	**Bin.27_lim**	81.2	1.9	3.4	6337	37.4	142 977	0.2	3430	1	0.0	Genus *Fusibacter_C*
	**Bin.23_lim**	96.2	1.1	2.4	106 442	31.2	461 252	0.7	1972	3	0.0	Order *Bacteroidales*
	**Bin.22_lim**	79.2	1.1	1.3	5309	41.6	53 843	0.1	1457	*-*	*0.0*	Family *Erysipelotrichaceae*
	**Bin.18_lim**	97.3	0.0	2.4	37 598	34.4	140 114	0.2	2000	*-*	*0.0*	Family *Paludibacteraceae*
	***unbinned***	*-*	*-*	14.9	5762	*-*	16 727 548	25.9	15 000	334	29.5	-

Beyond the genetic fingerprints of the two cultures, we analysed the proteomes of the same samples to quantify the relative biomass contribution and infer the actual metabolic role of each microorganism represented by a MAG [61]. Per sample, an average of 20 981 and 16 832 MS2 spectra (*N_2_Oexc* and *N_2_Olim*, respectively) was obtained over quadruplicate injections. We identified an average of 14 490 and 12 449 peptides, resulting in 1613 and 1324 proteins identified with a FDR < 0.01 by at least 2 unique peptides in at least 2 out of 4 injections. The relative abundance of a protein was calculated by dividing its normalized spectral counts by the sum of the normalized spectral counts in the corresponding injection. About 91.7 and 74.7% of the proteins identified in *N_2_Oexc* and *N_2_Olim*, respectively, were mapped to the recovered MAGs, providing a comprehensive coverage of the expressed functional potential of the enrichments. AZO_exc and AZO_lim accounted for 70.1 and 50.6% of their respective metaproteome, consistent with their metagenomic-based relative abundance (Table 2). In contrast, THA_lim, that represented only 1.9% of the metagenomic reads in *N_2_Olim*, accounted for 14.0% of the metaproteome, suggesting a more prominent metabolic role than predicted based on the sole metagenome. Moreover, for the three dominant MAGs we identified 1077 (AZO_exc), 648 (AZO_lim), and 181 (THA_lim) proteins representing the 41.2, 26.0, and 4.8% of their respective metagenome-predicted coding genes.

Taxonomically, the recovered draft genomes were affiliated to the phyla *Bacteroidota* (*Bacteroidetes*; 12), *Pseudomonadota* (*Proteobacteria*; 7), *Bacillota (Firmicutes;* 5), *Bdellovibrionota* (1), *Patescibacteria* (1), and *Deferribacterota* (1) (Table 2; Table S2). All three dominant MAGs (AZO_exc, AZO_lim, and THA_lim) affiliated with the family *Rhodocyclaceae*, and shared an average nucleotide identity <84% indicating that they do not belong to the same species [62] (see Table S2). AZO_exc and AZO_lim affiliated with the genus *Azonexus*, while THA_lim with the genus *Thauera*. The selectivity of the imposed conditions is further supported by 16S rRNA gene-based amplicon sequencing (Fig. S3). A single OTU affiliated with the *Rhodocyclaceae* family, initially present at very low relative reads abundances in the inoculum (< 1%), was progressively enriched in *N_2_Oexc*. The OUT reached steady-state relative abundances between 55 and 70%, in strong agreement with the AZOexc abundance in the metagenome. Two OTUs affiliated with the *Rhodocyclaceae* family dominated *N_2_Olim*, consistently with the steady-state metagenomic coexistence of AZO_lim and THA_lim. Globally, the microbially diverse and metabolically versatile *Rhodocyclaceae* family constitutes a core taxon in WWTP microbiomes, driving denitrification and likely N_2_O reduction across diverse ecosystems [9, 12, 63–65]. *Rhodocyclaceae* members are also commonly co-enriched based on their N_2_O reducing capability under non-axenic conditions in chemostats [28, 29, 66, 67] and biofilm-based systems [15]. Within the *Rhodocyclaceae* family, *Azonexus* genus affiliates have recently been enriched and isolated from biogas digestate, and their catabolic preference for N_2_O reduction has been shown both at kinetic and proteomic level [68]. Our study further supports the prominent role of *Rhodocyclaceae* family members in providing a potential N_2_O sink in diverse ecosystems.

### High-affinity clade II N_2_O-reducers selected under N_2_O limitation and low biomass dilution rates

**Figure 2:**
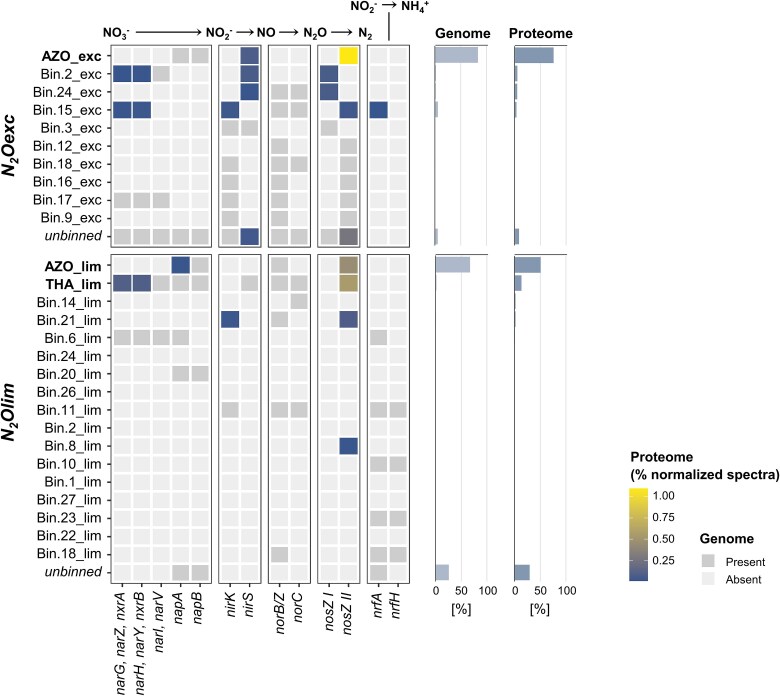
Denitrification and dissimilatory nitrite reduction to ammonium (DNRA). Gene presence (dark grey tiles) and protein abundance (colored tiles) for the high- and medium-quality (completeness >75% and contamination <5%) MAGs enriched under excess (*N_2_Oexc*) and limiting (*N_2_Olim*) nitrous oxide availability. The sum of the normalized spectral counts for each protein was taken as a proxy for protein abundance. Right bar charts: relative abundance of each MAG in the metagenome (based on relative reads alignment) and the metaproteome (summed relative abundance of normalized spectral counts of peptides matching to predicted proteins in each MAG). Abbreviations: *napAB*: Electron transfer (*napB*; EC:1.9.6.1; K02567) and catalytic (*napA*; K02568) subunits of the periplasmic nitrate reductase. *narGHI*: membrane-bound nitrate reductase, comprising the transmembrane protein *narI* (EC:1.7.5.1, 1.7.99.-; K00374) mediating the electron transfer from the quinol pool via *narH* (EC:1.7.5.1, 1.7.99.-; K00371) to the catalytic subunit *narG* (EC:1.7.5.1, 1.7.99.-; K00370). *nirK*: copper-containing nitrite reductase (EC:1.7.2.1; K00368). *nirS*: cytochrome *cd_1_*-containing nitrite reductase (EC:1.7.2.1, 1.7.99.1; K15864). *norCB*: nitric oxide reductase comprising the two subunits *norC* (K02305) and *norB/Z*; the latter comprises both the cytochrome c-dependent (*cNorB*; EC:1.7.2.5; K04561) and the menaquinol-dependent (*qNorB*/*NorZ*; EC:1.7.5.2) variants [68]. *nrfAH*: catalytic (*nrfA*; EC:1.7.2.2; K03385) and electron donating (*nrfH*; K15876) subunits of the periplasmic dissimilatory cytochrome c nitrite reductase.

We interrogated the recovered MAGs for genes and proteins involved in the reduction of nitrogen oxides. Consistently with nitrous oxide being the sole electron acceptor, the nitrous oxide reductase (NosZ) was among the ten most abundant proteins with an assigned KO number in *N_2_Oexc* and *N_2_Olim*, accounting for 1.4 and 1.1% of the total normalized spectral counts respectively (Fig. 2; Table S2). All recovered MAGs under N_2_O excess possessed a *nosZ* gene. AZO_exc harbored genes encoding for the clade II NosZ protein, and contributed the most to its overall expression (Fig. 2). Clade I harboring genomes were also present in *N_2_Oexc*, albeit at very low relative abundances (Fig. 2). The co-existence of the two clades aligns with the majority of characterized natural communities [4, 69] and N_2_O-respiring enrichments [15, 26, 27, 29, 67], and suggests that the imposed conditions were not selective enough to fully outcompete clade I N_2_O-reducers. In contrast, N_2_O limitation resulted in the complete washout of the clade I N_2_O-respiring microorganisms initially present in the inoculum (i.e. *N_2_Oexc*), as further confirmed by the absence of the clade I *nosZ* gene sequence even within the unbinned reads (Fig. 2). The NosZ protein belonging to the most abundant AZO_lim and THA_lim shared about 80% of sequence identity with the NosZ of AZO_exc (*N_2_Oexc*). Overall, while clade II N_2_O-reducers clearly played a primary role in both enrichments, to the best of our knowledge *N_2_Olim* constitutes the first enrichment of sole clade II *nosZ* harboring organisms in mixed microbial communities.

Contrary to our expectations, in terms of other reductases, all *nosZ* encoding genomes were not specialist N_2_O-reducers and possessed at least another nitrate or nitrite reductase gene. Clade II *nosZ* was annotated alone only in the low-abundant medium-quality (76.4% completeness) Bin.8_lim (Fig. 2). The periplasmic nitrate reductase (*napAB*) was encoded by all three dominant MAGs (AZO_exc, AZO_lim, THA_lim), while only THA_lim also possessed the membrane-bound dissimilatory nitrate reductase *narGHI*. In agreement with published denitrifiers genomes [9], MAGs possessing the cytochrome *cd_1_*-type nitrite reductase (*nirS*) did not encode the copper-containing nitrite reductase (*nirK*), and vice versa, with the only exception of the low-abundant Bin.3_exc encoding both (*N_2_Oexc*). Yet, none of the *nir* was annotated in AZO_lim (*N_2_Olim*). In contrast to often observed co-occurrence patterns [4, 9], no evident correlation emerged between the presence of *nirS* and *nosZ*, or the nitric oxide reductase (*cNorB* or *qNorB*). The ammonification potential, identified by the presence of the periplasmic energy-conserving periplasmic cytochrome c nitrite reductase *nrfAH* complex, was almost exclusively restricted to the N_2_O-limiting conditions with acetate excess (*N_2_Olim*), in an interesting parallel with the electron donor-rich environments selecting for ammonifiers [70]. Despite N_2_O being the only electron acceptor, some NarG/NapA and NirK/NirS enzymes were detected in the proteome, yet at more than one order of magnitude lower abundances than NosZ (Fig. 2). The observed constitutive expression of the denitrifying enzymes is consistent with the significant maximum *ex-situ* nitrite reduction capacity maintained in both enrichments (Fig. 1) in analogy to [29] and [25].

Taken together, our results suggest that N_2_O limiting conditions at sufficiently low growth rates (here 0.006 h^−1^) exclusively select for *clade II* N_2_O-reducers. To date, this was only hypothesized based on the higher affinity displayed by a limited number of characterized clade II isolates [15, 16], and the positive correlation between clade II *nosZ* gene abundance and long retention times reported in the work of Conthe and colleagues [28, 29]. Follow-up research should target the independent experimental confirmation of our findings and the currently elusive conditions that further select for non-denitrifying specialist N_2_O-reducers in natural and engineered microbiomes.

### Cobalamin auxotrophy as potential competitive advantage at high N_2_O

We hypothesize that the cytotoxic effects of elevated N_2_O levels in the growth medium led to the observed lower growth yields in *N_2_Oexc*, and shaped the corresponding community assembly. N_2_O is known to selectively inactivate cobalamin (vitamin B_12_), an essential cofactor in bacterial metabolism [33, 59]. High N_2_O concentrations have been shown to hinder microbial growth by compromising the activity of cobalamin-dependent enzymes [60]. Cobalamin synthesis itself constitutes a significant genetic and metabolic burden for the cell, involving up to thirty enzymes [71], and in environmental communities it is usually performed by a small cohort of prototrophic low-abundant microorganisms [72, 73]. In this perspective, genes encoding proteins involved in cobalamin synthesis and transport, and in core cobalamin-dependent metabolic pathways were searched for in the recovered MAGs.

**Figure 3:**
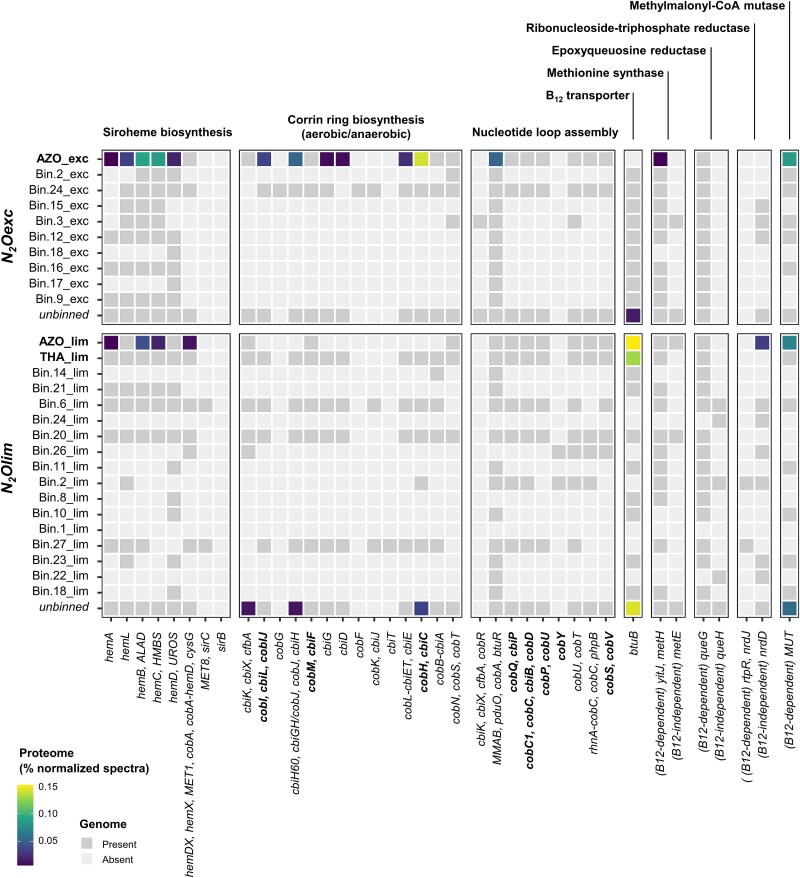
Cobalamin bio-synthesis and transport, and cobalamin (in)dependent enzymes. Gene presence (dark grey tiles) and protein abundance (colored tiles) for the high- and medium-quality MAGs enriched under excess (*N_2_Oexc*) and limiting (*N_2_Olim*) nitrous oxide availability. Annotations: corrin ring biosynthesis (aerobic/anaerobic) marker genes: *cobI*, *cbiL*, *cobIJ* (EC:2.1.1.130, EC:2.1.1.151; K03394, K13540). *cub*, *cbiF* (EC:2.1.1.133, EC:2.1.1.271: K05936). cobH, cbiC (EC:5.4.99.61, EC:5.4.99.60; K06042). Nucleotide loop assembly marker genes: *cobQ*, *cbiP (EC:*6.3.5.10; K02232). *cobC1*, *cobC*, *cbiB*, *cobD* (EC:6.3.1.10; K02225, K02227). *cobP*, *cobU* (EC:2.7.1.156; K02231). *cobS*, *cobV* (EC:2.7.8.26; K02233). *cobY* (EC:2.7.7.62; K19712). Transport and (in)dependent enzymes. *btuB*: cobalamin/cobinamide transporter (K16092). *yitJ*, *metH*: cobalamin-dependent methionine synthase (EC:2.1.1.13, EC:1.5.1.54; K00548, K24042). *metE*: cobalamin-independent methionine synthase (EC:2.1.1.14; K00549). *queG*: cobalamin-dependent epoxyqueuosine reductase (EC:1.17.99.6; K18979). *queH*: cobalamin-independent epoxyqueuosine reductase (EC:1.17.99.6; K09765). *nrdJ*, *rtpR*: cobalamin-dependent ribonucleoside-triphosphate reductase (EC:1.17.4.2; K00524, K00527). *nrdD*: cobalamin-independent ribonucleoside-triphosphate reductase (EC:1.1.98.6; K21636). *MUT*: methylmalonyl CoA mutase (EC:5.4.99.2; K01847). Details for all bio-synthesis genes can be found in Table S3.

We identified AZO_exc (*N_2_Oexc*) and THA_lim (*N_2_Olim*) as de novo cobalamin producers based on the presence of seven of the eight experimentally-verified biosynthetic marker genes proposed by [73] (corrin ring biosynthesis: *cbiL*/*cobI*, *cbiF*/*cobM*, *cbiC*/*cobH*; nucleotide loop assembly: *cobQ/cbiP*, *cobC1/cobC/cbiB/cobD*, *cobS/cobV*, and *cobP/cobU*, except *cobY*; Fig. 3, Table S3). The entire cobalamin production pathway was also almost completely annotated in both MAGs, and the majority of predicted proteins were detected in AZO_exc. In contrast, AZO_lim (*N_2_Olim*) contained all nucleotide loop assembly steps, but lacked two of the downstream corrin ring biosynthetic marker genes, namely *cbiL*/*cobI* and *cbiC*/*cobH*, along with a more inconsistent pathway annotation (Fig. 3). On these grounds, while missing annotations e.g. due to the lower MAG completeness (87.3%) or non-homologous replacements cannot be ruled out, AZO_lim seems to have relied on the uptake of late cobalamin intermediates (e.g. cobinamide) either from the yeast extract present in the influent or produced by THA_lim [73]. The high protein expression of the outer membrane cobalamin/cobinamide transporter (BtuB; [71, 72]) in both MAGs further supports this hypothesis (Fig. 3). Conversely, in *N_2_Oexc*, AZO_exc was devoid of the vitamin B12 transporter gene *btuB*, and the BtuB protein was detected at low abundances only in side populations.

Both enrichments encoded the cobalamin-dependent processes most widely found in bacterial genomes, encompassing amino acid and nucleotide synthesis [72, 73]. All three dominant MAGs possessed the cobalamin-dependent methionine synthase (*metH*), while AZO_lim encoded also its cobalamin-independent orthologue (*metE*; Fig. 3). *metE* has been previously shown to be up-regulated under cobalamin-limiting conditions, e.g. at high N_2_O, albeit displaying significantly lower activities [60]. Yet, only the cobalamin-dependent MetH was detected in the proteome of AZO_lim. The reliance of all three MAGs on vitamin B12 was further supported by the presence of the cobalamin-dependent epoxyqueuosine reductase (*queG*), involved in tRNA modification, and by the expression of methylmalonyl CoA mutase (*MUT*), catalyzing the synthesis of succinyl-CoA [74].

We suggest that de novo cobalamin production provided AZO_exc with a selective advantage in *N_2_Oexc*. High N_2_O concentrations likely compromised both the internal and exogenous (i.e. from yeast extract) active vitamin B12 pool, making cobalamin self-sufficiency an essential metabolic trait. Conversely, the more permissive conditions in *N_2_Olim* made cobalamin cross-feeding viable, and its production dispensable [75]. The high expression of the cobalamin transporter (BtuB) may suggest the reliance of both AZO_lim and THA_lim on the uptake of the exogenous yeast extract cobalamin. However, we reason that the genome-inferred cobalamin-prototrophy provided AZO_lim with a higher relative fitness, i.e. allowing for an optimized cellular proteome allocation [76–78], while creating the ecological niche for the unexpected co-enrichment of the putative cobalamin-producer THA_lim. Overall, the abundances of the corresponding proteins were relatively low especially in the *N_2_Olim* (Fig. 3), in line with reported low detections of cobalamin production gene-transcripts even in cobalamin-free cultures [79]. While caution is warranted in their interpretation, and pending direct experimental confirmation, the higher detection of cobalamin synthesis proteins in *N_2_Oexc* compared to *N_2_Olim* (Fig. 3) further emphasizes an increased need to sustain an otherwise continuously compromised cobalamin pool. Ultimately, our genome-resolved metaproteome-based evidence suggests that cobalamin cross-feeding may partially underpin the often reported coexistence of N_2_O reducers in natural and engineered complex ecosystems.

## Supplementary Material

250307_N2O_B12_ISMEcomm_SI_CLEAN_ycaf022

## Data Availability

Raw DNA reads were deposited on the NCBI Sequence Read Archive and medium- and high-quality MAGs were deposited in Genbank under BioProject PRJNA1054980, with MAG accession numbers SAMN46430372 - SAMN46430381 for *N_2_Oexc*, and SAMN46430382 - SAMN46430398 for *N_2_Olim*, in the same order as in Table 2. The 16S rRNA gene amplicon sequencing data can be found under BioProject PRJEB84459. The mass spectrometry proteomics raw data, reference sequence database and database search files have been deposited in the ProteomeXchange consortium database with the dataset identifier PXD030677.
