# Supplementary material for: Selective enrichment of high-affinity clade II N2O-reducers in a mixed culture
Source: ISME Commun. 2025 Feb 5;5(1):ycaf022. doi: 10.1093/ismeco/ycaf022 (PMC11906303; doi:10.1093/ismeco/ycaf022)
Supplement: 250307_N2O_B12_ISMEcomm_SI_CLEAN_ycaf022 [file 250307_n2o_b12_ismecomm_si_clean_ycaf022.pdf]

Supporting information

Selective enrichment of high-affinity clade II N_2O-reducers in a mixed culture

Michele Laureni[1*], Francesc Corbera-Rubio[1], DaeHyun Daniel Kim[2,a], Savanna Browne[1], Nina Roothans[1], David G. Weissbrodt[3], Karel Olavaria[1], Nadieh de Jonge[4], Sukhwan Yoon[2], Martin Pabst[1], Mark C.M. van Loosdrecht[1]

[1] Department of Biotechnology, Delft University of Technology, Van der Maasweg 9, Delft, HZ NL- 2629, The Netherlands
[2] Department of Civil and Environmental Engineering, Korea Advanced Institute of Science and Technology, Daehakro 291, KAIST, Daejeon 34141, South Korea
[3] Department of Biotechnology and Food Science, Norwegian University of Science and Technology, Sem Sælands vei 8, 7034 Trondheim, Norway
[4] Department of Chemistry and Bioscience, Aalborg University, Fredrik Bajers Vej 7H, DK-9220 Aalborg, Denmark
[a] current address: Department of Civil and Environmental Engineering, University of California, Berkeley, CA, USA

* Corresponding author: *Michele Laureni*. Current address, Department of Water Management, Delft University of Technology, Stevinweg 1, 2628 CN Delft, the Netherlands. (m.laureni@tudelft.nl)

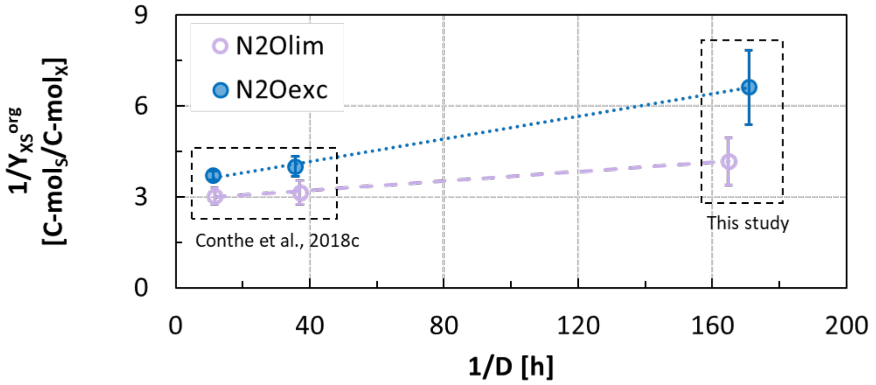

Figure S1. Estimation of the biomass-specific acetate consumption rate for maintenance based on the estimated growth yields on acetate over the dilution rates range covered by this study and (Conthe et al. 2018).

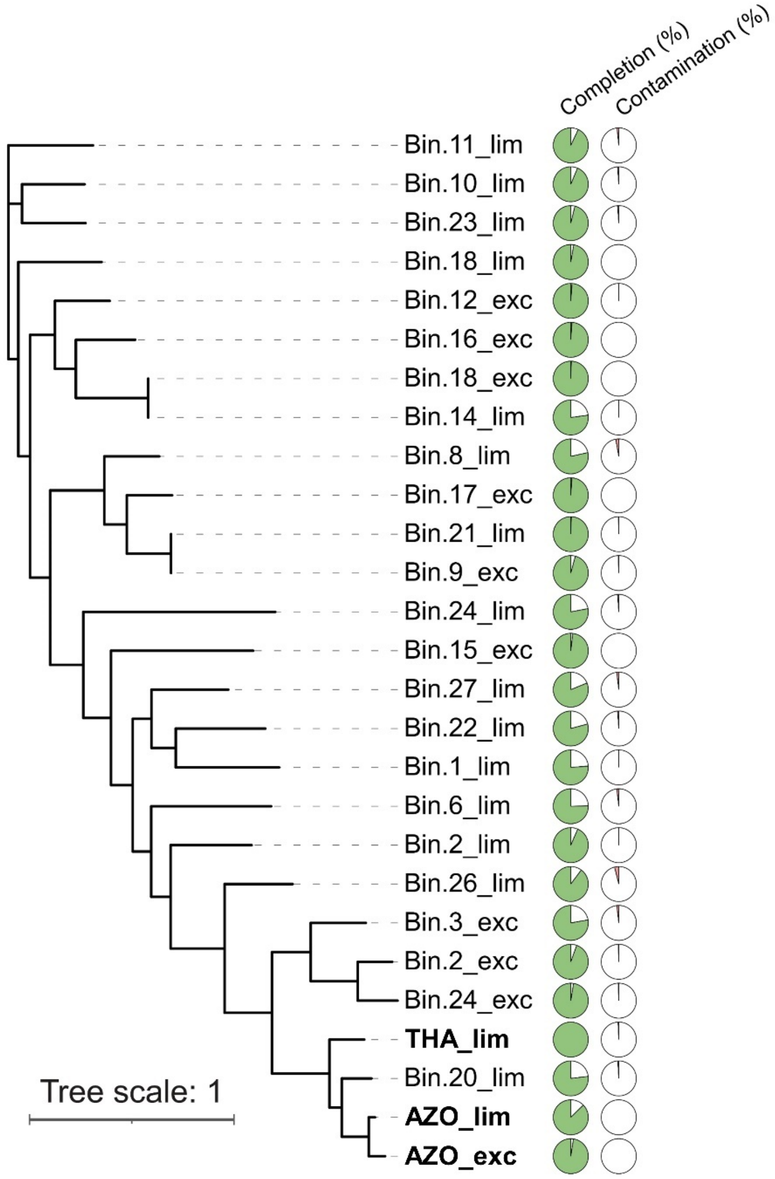

Figure S2. Genomic phylogenetic tree of the 27 MAGs, including the corresponding completeness and contamination values. The software Anvi'o v.8 was used to align concatenated ribosomal genes identified across MAGs. The tree was built from the alignment using FastTree with the generalized time-reversible model, and the visual output was generated using the iTOL v.6.6 software.

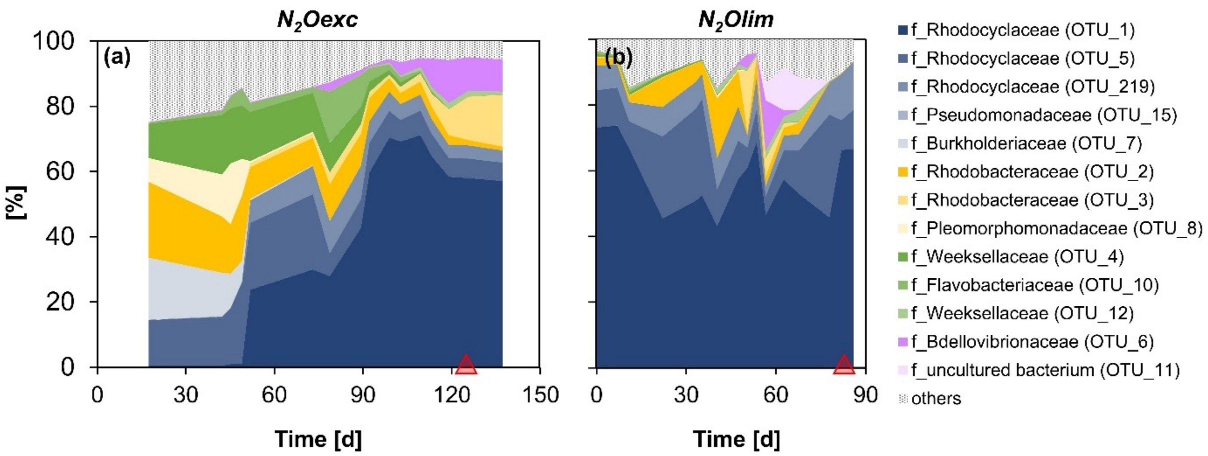

Figure S3. Microbial composition dynamics of *N_2Oexc* and *N_2Olim* based on 16S rRNA gene-based amplicon sequencing. The most abundant Operational Taxonomic Units (OTUs), with a relative reads abundance > 10 % in at least one sample, are presented at family level. Triangles mark the metagenomic and metaproteomic sampling day. *DNA extraction and amplicon sequencing.* Biomass samples were collected on a weekly basis for amplicon sequencing. Total DNA was extracted using FastDNA Spin Kit for Soil (MP Biomedicals). Extracts were quantified using the Qubit dsDNA Broad Range Assay Kit and a Qubit 3.0 fluorometer (Thermo Fisher Scientific), and normalized to 5 ng·µL^{-1} prior to amplification. The relative microbial compositions was determined by amplifying the V3-V4 region of the 16S rRNA gene of 10 ng of genomic DNA with the V3-V4 primer from (Takahashi et al. 2014). The total reaction volume of 25 µL contained 2 mU Platinum Taq DNA Polymerase, 1× Platinum High Fidelity buffer (Thermo Fisher Scientific), 400 nM of each dNTP, 1.5 mM $MgSO_4$, and 400 nM of each primer fused with Illumina adaptors. Amplicons were validated with Qubit dsDNA High Sensitivity Assay Kit (Thermo Fisher Scientific), and TapeStation 2200 with D1000 ScreenTapes (Agilent). Amplicons were purified using Ampure XP bead protocol (Beckmann Coulter) with a bead:sample ratio of 0.8. Barcoding was performed according to the Nextera XT barcode protocol (Illumina). Libraries were pooled in equimolar concentrations and subsequently sequenced on a MiSeq platform (Illumina) using MiSeq reagent kit v3 (2 × 300 PE), and a 20% PhiX spike-in. *Data processing and analysis.* Raw sequencing reads were quality controlled using Trimmomatic v0.32 (Bolger et al. 2014), and subsequently merged with FLASH v1.2.7 (Magoc and Salzberg 2011). After being screened for PhiX contamination, reads were formatted for use using the UPARSE pipeline (Edgar 2013). USEARCH7 was used to remove chimeric sequences and clustering into Operational Taxonomic Units (OTUs) at 97% sequence similarity. Taxonomy was assigned using the RDP algorithm implemented in QIIME (Caporaso et al. 2010). The SILVA database release S132 was used as reference (Quast et al. 2013). R version 3.5.2 (https://cran.r-project.org/) via RStudio version 1.1.463 (https://www.rstudio.com/) and the ampvis2 package (Andersen et al. 2018) were used for sequencing data analysis. The number of high-quality reads per sample varied between 12505 and 45507. Rarefaction curves showed that the sequencing depth sufficiently covered species richness in all samples.

Table S1. Average and standard deviation of the dilution rate (D), and the conversion rates for the two enrichments. Values are calculated from the steady-state reactor operation measurements (Figure 1; days 100-150 for *N_2Oexc* and 60-100 for *N_2Olim*). The software Macrobal (Hellinga and Romein 1992) was used for data reconciliation.

**D**	**Acetate**	**N2O**	**NH4+**	**X**	**H+**	
**h^{-1}**	**mmol/d**	**mmol/d**	**mmol/d**	**mmol/d**	**mmol/d**	
0.0059 ± 0.001	-30.6 ± 1.8	-104.7 ± 8.8	-3.8 ± 1.2	9.3 ± 1.6	-26.8 ± 2	**N_2Oexc**
0.0061 ± 0.0007	-24.4 ± 1.1	-75.6 ± 1.6	-5.1 ± 1.2	11.7 ± 2.1	-19.2 ± 1.5	**N_2Olim**

Table S2. Full taxonomy of the recovered bins. MAGs marked with (ǁ) or (‡) share a genome-aggregate average nucleotide identity (ANI) > 99 %.

	Bin ID	Phylum	Class	Order	Family	Genus	Species
N_2Oexc	**AZO_exc**	Pseudomonadota	Gammaproteobacteria	Burkholderiales	Rhodocyclaceae	Azonexus	
	**Bin.2_exc**	Pseudomonadota	Alphaproteobacteria	Rhodobacterales	Rhodobacteraceae	Phaeovulum	
	**Bin.24_exc**	Pseudomonadota	Alphaproteobacteria	Rhodobacterales	Rhodobacteraceae	Pseudorhodobacter	
	**Bin.15_exc**	Bdellovibrionota	Bdellovibrionia	Bdellovibrionales	Bdellovibrionaceae	Bdellovibrio	Bdellovibrio sp019104905
	**Bin.3_exc**	Pseudomonadota	Alphaproteobacteria	Rhizobiales	Rhizobiaceae	Aquamicrobium	
	**Bin.12_exc**	Bacteroidota	Bacteroidia	Flavobacteriales	Flavobacteriaceae	Flavobacterium	Flavobacterium filum
	**Bin.18_exc**[‡]	Bacteroidota	Bacteroidia	Flavobacteriales	Weeksellaceae	Moheibacter	Moheibacter sp019455045
	**Bin.16_exc**	Bacteroidota	Bacteroidia	Flavobacteriales	Weeksellaceae	Kaistella	
	**Bin.17_exc**	Bacteroidota	Bacteroidia	Chitinophagales	Chitinophagaceae	Ferruginibacter	Ferruginibacter sp001898465
	**Bin.9_exc**[ǁ]	Bacteroidota	Bacteroidia	Chitinophagales	Chitinophagaceae	Ferruginibacter	Ferruginibacter sp018268175
	unbinned						
N_2Olim	**AZO_lim**	Pseudomonadota	Gammaproteobacteria	Burkholderiales	Rhodocyclaceae	Azonexus	Azonexus sp900549295
	**THA_lim**	Pseudomonadota	Gammaproteobacteria	Burkholderiales	Rhodocyclaceae	Thauera	Thauera phenylacetica
	**Bin.14_lim**[‡]	Bacteroidota	Bacteroidia	Flavobacteriales	Weeksellaceae	UBA3376	
	**Bin.21_lim**[ǁ]	Bacteroidota	Bacteroidia	Chitinophagales	Chitinophagaceae	Ferruginibacter	Ferruginibacter sp018268175
	**Bin.6_lim**	Deferribacterota	Deferribacteres	Deferribacterales	Denitrovibrionaceae	Seleniivibrio	Seleniivibrio woodruffii
	**Bin.24_lim**	Patescibacteria	JAEDAM01	BD1-5	UBA6164	UBA7396	
	**Bin.20._lim**	Pseudomonadota	Gammaproteobacteria	Burkholderiales	Rhodocyclaceae	Azovibrio	Azovibrio restrictus
	**Bin.26_lim**	Bacillota	Clostridia	Oscillospirales	Oscillospiraceae	Pseudoflavonifractor	
	**Bin.11_lim**	Bacteroidota	Bacteroidia	Bacteroidales	F082	JALNZU01	JALNZU01 sp002421995
	**Bin.2_lim**	Bacillota	Clostridia	Peptostreptococcales	Anaerovoracaceae	UBA5559	
	**Bin.8_lim**	Bacteroidota	Bacteroidia	Chitinophagales	Chitinophagaceae	Niabella	
	**Bin.10_lim**	Bacteroidota	Bacteroidia	Bacteroidales	WCHB1-69	UBA5429	
	**Bin.1_lim**	Bacillota	Bacilli	Acholeplasmatales	UBA5453	Paracholeplasma	
	**Bin.27_lim**	Bacillota	Clostridia	Peptostreptococcales	Acidaminobacteraceae	Fusibacter_C	Fusibacter_C sp001897605
	**Bin.23_lim**	Bacteroidota	Bacteroidia	Bacteroidales	P3	UBA10566	UBA10566 sp002399565
	**Bin.22_lim**	Bacillota	Bacilli	Erysipelotrichales	Erysipelotrichaceae	UBA2212	
	**Bin.18_lim**	Bacteroidota	Bacteroidia	Bacteroidales	Paludibacteraceae	UPXZ01	
	unbinned						

Table S3. Annotation of the enzymes involved in cobalamin synthesis and transport, and the cobalamin-dependent enzymes with their cobalamin-independent functional homologues.

ko	ko_name	EC_1	EC_2	EC_3	name	pathway
K02492	hemA	1.2.1.70			hemA	Siroheme biosynthesis
K01845	hemL	5.4.3.8			hemL	Siroheme biosynthesis
K01698	hemB, ALAD	4.2.1.24			hemB, ALAD	Siroheme biosynthesis
K01749	hemC, HMBS	2.5.1.61			hemC, HMBS	Siroheme biosynthesis
K01719	hemD, UROS	4.2.1.75			hemD, UROS	Siroheme biosynthesis
K02302	cysG	2.1.1.107	1.3.1.76	4.99.1.4	hemDX, hemX, MET1, cobA, cobA-hemD, cysG	Siroheme biosynthesis
K02303	cobA	2.1.1.107			hemDX, hemX, MET1, cobA, cobA-hemD, cysG	Siroheme biosynthesis
K13542	cobA-hemD	2.1.1.107	4.2.1.75		hemDX, hemX, MET1, cobA, cobA-hemD, cysG	Siroheme biosynthesis
K00589	MET1	2.1.1.107			hemDX, hemX, MET1, cobA, cobA-hemD, cysG	Siroheme biosynthesis
K02496	hemX	2.1.1.107			hemDX, hemX, MET1, cobA, cobA-hemD, cysG	Siroheme biosynthesis
K13543	hemDX	2.1.1.107	4.2.1.75		hemDX, hemX, MET1, cobA, cobA-hemD, cysG	Siroheme biosynthesis
K02304	MET8	1.3.1.76	4.99.1.4		MET8, sirC	Siroheme biosynthesis
K24866	sirC	1.3.1.76			MET8, sirC	Siroheme biosynthesis
K03794	sirB	4.99.1.4			sirB	Siroheme biosynthesis
K02190	cbiK	4.99.1.3			cbiK, cbiX, cfbA	Corrin ring biosynthesis (aerobic/aerobic)
K03795	cbiX	4.99.1.3			cbiK, cbiX, cfbA	Corrin ring biosynthesis (aerobic/aerobic)
K22011	cfbA	4.99.1.3	4.99.1.11		cbiK, cbiX, cfbA	Corrin ring biosynthesis (aerobic/aerobic)
K03394	cobI-cbiL	2.1.1.130	2.1.1.151		cobI-cbiL, cobIJ	Corrin ring biosynthesis (aerobic/aerobic)
K13540	cobIJ	2.1.1.130	2.1.1.131		cobI-cbiL, cobIJ	Corrin ring biosynthesis (aerobic/aerobic)
K02229	cobG	1.14.13.83			cobG	Corrin ring biosynthesis (aerobic/aerobic)
K05934	cobJ, cbiH	2.1.1.272	2.1.1.131		cbiH60, cbiGH-cobJ, cobJ, cbiH	Corrin ring biosynthesis (aerobic/aerobic)
K13541	cbiGH-cobJ	2.1.1.272	2.1.1.131	3.7.1.12	cbiH60, cbiGH-cobJ, cobJ, cbiH	Corrin ring biosynthesis (aerobic/aerobic)
K21479	cbiH60	2.1.1.272			cbiH60, cbiGH-cobJ, cobJ, cbiH	Corrin ring biosynthesis (aerobic/aerobic)
K05936	cobM, cbiF	2.1.1.133	2.1.1.271		cobM, cbiF	Corrin ring biosynthesis (aerobic/aerobic)
K02189	cbiG	3.7.1.12			cbiG	Corrin ring biosynthesis (aerobic/aerobic)
K02188	cbiD	2.1.1.195			cbiD	Corrin ring biosynthesis (aerobic/aerobic)
K02228	cobF	2.1.1.152			cobF	Corrin ring biosynthesis (aerobic/aerobic)
K05895	cobK-cbiJ	1.3.1.54	1.3.1.106		cobK-cbiJ	Corrin ring biosynthesis (aerobic/aerobic)
K02191	cbiT	2.1.1.196			cbiT	Corrin ring biosynthesis (aerobic/aerobic)
K00595	cobL-cbiET	2.1.1.289	2.1.1.196	2.1.1.132	cobL-cbiET, cbiE	Corrin ring biosynthesis (aerobic/aerobic)
K03399	cbiE	2.1.1.289			cobL-cbiET, cbiE	Corrin ring biosynthesis (aerobic/aerobic)
K06042	cobH-cbiC	5.4.99.61	5.4.99.60		cobH-cbiC	Corrin ring biosynthesis (aerobic/aerobic)
K02224	cobB-cbiA	6.3.5.9	6.3.5.11		cobB-cbiA	Corrin ring biosynthesis (aerobic/aerobic)
K02230	cobN	6.6.1.2			cobN, cobS, cobT	Corrin ring biosynthesis (aerobic/aerobic)
K09882	cobS	6.6.1.2			cobN, cobS, cobT	Corrin ring biosynthesis (aerobic/aerobic)

K09883	cobT	6.6.1.2		cobN, cobS, cobT	Corrin ring biosynthesis (aerobic/aerobic)
K13786	cobR			cobR	Nucleotide loop assembly
K00798	MMAB, pduO	2.5.1.17		MMAB, pduO, cobA, btuR	Nucleotide loop assembly
K19221	cobA, btuR	2.5.1.17		MMAB, pduO, cobA, btuR	Nucleotide loop assembly
K02232	cobQ, cbiP	6.3.5.10		cobQ, cbiP	Nucleotide loop assembly
K02225	cobC1, cobC	6.3.1.10		cobC1, cobC, cbiB, cobD	Nucleotide loop assembly
K02227	cbiB, cobD	6.3.1.10		cobC1, cobC, cbiB, cobD	Nucleotide loop assembly
K02231	cobP, cobU	2.7.1.156		cobP, cobU	Nucleotide loop assembly
K19712	cobY	2.7.7.62		cobY	Nucleotide loop assembly
K00768	cobU, cobT	2.4.2.21		cobU, cobT	Nucleotide loop assembly
K02226	cobC, phpB	3.1.3.73		rh-cobC, cobC, phpB	Nucleotide loop assembly
K22316	rh-cobC	3.1.3.73	3.1.26.4	rh-cobC, cobC, phpB	Nucleotide loop assembly
K02233	cobS, cobV	2.7.8.26		cobS, cobV	Nucleotide loop assembly
K16092	btuB			btuB	B12 transporter
K00548	metH, MTR	2.1.1.13		(B12-dependent) yitJ, metH	Methionine synthase
K24042	yitJ	2.1.1.13	1.5.1.54	(B12-dependent) yitJ, metH	Methionine synthase
K00549	metE	2.1.1.14		(B12-independent) metE	Methionine synthase
K18979	queG	1.17.99.6		(B12-dependent) queG	Epoxyqueuosine reductase
K09765	queH	1.17.99.6		(B12-independent) queH	Epoxyqueuosine reductase
K00524	nrdJ	1.17.4.2		(B12-dependent) rtpR, nrdJ	Ribonucleoside-triphosphate reductase
K00527	rtpR	1.17.4.2		(B12-dependent) rtpR, nrdJ	Ribonucleoside-triphosphate reductase
K21636	nrdD	1.1.98.6		(B12-independent) nrdD	Ribonucleoside-triphosphate reductase
K01847	MUT	5.4.99.2		(B12-dependent) MUT	Methylmalonyl-CoA mutase

References

Andersen, K.S., Kirkegaard, R.H., Karst, S.M. and Albertsen, M. (2018) ampvis2: an R package to analyse and visualise 16S rRNA amplicon data. bioRxiv https://doi.org/10.1101/299537

Bolger, A.M., Lohse, M. and Usadel, B. (2014) Trimmomatic: a flexible trimmer for Illumina sequence data. Bioinformatics 30(15), 2114-2120.

Caporaso, J.G., Kuczynski, J., Stombaugh, J., Bittinger, K., Bushman, F.D., Costello, E.K., Fierer, N., Peña, A.G., Goodrich, J.K., Gordon, J.I., Huttley, G.A., Kelley, S.T., Knights, D., Koenig, J.E., Ley, R.E., Lozupone, C.A., McDonald, D., Muegge, B.D., Pirrung, M., Reeder, J., Sevinsky, J.R., Turnbaugh, P.J., Walters, W.A., Widmann, J., Yatsunenko, T., Zaneveld, J. and Knight, R. (2010) QIIME allows analysis of high-throughput community sequencing data. Nat Methods 7(5), 335-336.

Conthe, M., Wittorf, L., Kuenen, J.G., Kleerebezem, R., van Loosdrecht, M.C.M. and Hallin, S. (2018) Life on N2O: deciphering the ecophysiology of N2O respiring bacterial communities in a continuous culture. ISME J 12(4), 1142-1153.

Edgar, R.C. (2013) UPARSE: highly accurate OTU sequences from microbial amplicon reads. Nat Methods 10(10), 996-998.

Hellinga, C. and Romein, B. (1992) MACROBAL: A Program for Robust Data Reconciliation and Gross Error Detection. IFAC Proceedings Volumes 25(2), 459-460.

Magoc, T. and Salzberg, S.L. (2011) FLASH: fast length adjustment of short reads to improve genome assemblies. Bioinformatics 27(21), 2957-2963.

Quast, C., Pruesse, E., Yilmaz, P., Gerken, J., Schweer, T., Yarza, P., Peplies, J. and Glockner, F.O. (2013) The SILVA ribosomal RNA gene database project: improved data processing and web-based tools. Nucleic Acids Res 41(Database issue), D590-596.

Takahashi, S., Tomita, J., Nishioka, K., Hisada, T. and Nishijima, M. (2014) Development of a prokaryotic universal primer for simultaneous analysis of Bacteria and Archaea using next-generation sequencing. PLoS One 9(8), e105592.
